# Twelve-Week Lower Trapezius-Centred Muscular Training Regimen in University Archers

**DOI:** 10.3390/healthcare10010171

**Published:** 2022-01-17

**Authors:** Chien-Nan Liao, Chun-Hao Fan, Wei-Hsiu Hsu, Chia-Fang Chang, Pei-An Yu, Liang-Tseng Kuo, Bo-Ling Lu, Robert Wen-Wei Hsu

**Affiliations:** 1 Department of Athletic Sports, National Chung Cheng University, Chia Yi 621, Taiwan; liao.cody@gmail.com; 2 Sports Medicine Center, Chang Gung Memorial Hospital, Chia Yi 621, Taiwan; fun711009@cgmh.org.tw (C.-H.F.); kojia882@yahoo.com.tw (C.-F.C.); b9002065@cgmh.org.tw (P.-A.Y.); light71829@gmail.com (L.-T.K.); bolin271@yahoo.com.tw (B.-L.L.); wwh@cgmh.org.tw (R.W.-W.H.); 3 Department of Orthopedic Surgery, Chang Gung Memorial Hospital, Chia Yi 621, Taiwan; 4 School of Medicine, Chang Gung University, Tao-Yuan 333, Taiwan

**Keywords:** electromyography, lower trapezius, muscle strength, shoulder kinematics

## Abstract

Archery is a fine-motor-skill sport, in which success results from multiple factors including a fine neuromuscular tuning. The present study hypothesised that lower trapezius specific training can improve archers’ performance with concomitant changes in muscle activity and shoulder kinematics. We conducted a prospective study in a university archery team. Athletes were classified into exercise and control groups. A supervised lower trapezius muscle training program was performed for 12 weeks in the exercise group. The exercise program focused on a lower trapezius-centred muscular training. Performance in a simulated game was recorded as the primary outcome, and shoulder muscle strength, kinematics, and surface electromyography were measured and analysed. In the exercise group, the average score of the simulation game increased from 628 to 639 after the training regimens (maximum score was 720), while there were no such increases in the control group. The lower trapezius muscle strength increased from 8 to 9 kgf after training regimens and shoulder horizontal abductor also increased from 81 to 93 body weight% for the exercise group. The upper/lower trapezius ratio decreased from 2.2 to 1.1 after training. The lower trapezius exercise training regimen could effectively improve the performance of an archer with a simultaneous increase in shoulder horizontal abductor and lower trapezius muscle strength.

## 1. Introduction

Archery is a fine-motor-skill sport, in which success is defined by the ability to shoot a target repeatedly with high precision and accuracy [1,2,3]. Recurve archery is an Olympic sport that requires concentration, extreme precision, upper body strength, and endurance [4]. An archery shot has three phases: the stance phase; the arming phase, during which the archer pulls the bowstring and pushes the bow; and the sighting phase, which involves the final stretching of the bow while focusing on the target [2]. Because of the high precision required, minimising movements during aiming allows for greater repeatability and consistency of this closed-loop skill [5]. Therefore, specific neuromuscular training is necessary for precise aiming during the sighting phase [6].

In the sighting phase, archers are required to maintain certain angles of shoulder abduction, horizontal extension, and elbow joint flexion for shorter duration [7]. Therefore, a series of movements are necessary during a shot, which require strong activity of the muscles attached to the shoulder girdle and upper extremity [8]. Archer performance is affected by the muscle activity of the shoulder girdle and upper extremity, especially scapular stability and lower trapezius (LT) muscle activity, on the drawing side [8,9,10,11,12]. Similarly, kinematic studies have revealed that decreased scapular elevation and abduction and increased scapular extension are closely related to better performance [8,10,12]. Therefore, LT strengthening can enhance an archer’s performance [8]. However, few studies have analysed the effect of LT-centred muscular training on overhead athletes’ performance [13,14]. Accordingly, the present study investigated the effect of LT-centred muscular training exercises on the muscle strength, kinematics, and performance of university archers. The study hypothesised that LT-centred muscular training can improve university archers’ performance with concomitant changes in muscle activity and shoulder kinematics.

## 2. Materials and Methods

### 2.1. Participants

This prospective study recruited participants from a university archery team, who attended a supervised LT-centred muscular training programme for 12 weeks from June 2020 to August 2020. All participants involved in the present study were recruited from a university archer team, which recruited students from high school because of their good performance. Performance in a simulated game was recorded as the primary outcome, and shoulder muscle strength, kinematics, and surface electromyography (EMG) were measured and analysed.

In previous archery surveys, most athletes were divided into different groups with the number of people in each group ranging from 6 to 10 [8,10,15,16,17]. Therefore, this study was expected to recruit 20 archery athletes, and finally, a total of fourteen archers (all of whom used recurve bows) participated in this study. All participants provided written informed consent before the study. They were all right handed; they used their right hand for the drawing movement during archery. The individuals were excluded if they (1) had a history of surgery to either shoulder, (2) had a shoulder muscle disease, or (3) had shoulder impingement syndrome. This study was approved by the Chang Gung Medical Foundation Institutional Review Board (IRB: 201800990B0). All participants provided written informed consent.

### 2.2. Study Design

According to the wishes of athletes, all participants were divided into exercise (3 male: 4 female) and control groups (2 male: 5 female). All participants underwent the routine exercise protocol (Table S1). The exercise group received additional exercise. The following anthropometric parameters were measured: height, body weight, shoulder muscle strength, and scores in the simulated championship. Shoulder kinematics and electromyography were simultaneously recorded during archery shots. All measurements were performed before and after a 12-week intervention.

### 2.3. Simulation Game

Moreover, the composition of participants or environmental factors, such as the location of and weather at the championship, may affect their competitive ranking or athletic performance [18,19]. Therefore, to better isolate the effect of exercise training on competition scores, we calculated the participants’ scores on a simulation game before and after the 12-week training programme. The simulation game requires the player to perform 72 arrows shot at a distance of 70 m, and the highest possible score was 720. All simulation games were held at standard archery fields under the same weather conditions.

### 2.4. Upper Limb Joint Angle Motion

Arrow shooting data were collected in an indoor biomechanics laboratory. After a regular warm-up, each participant shot six arrows towards a target placed at a 5 m distance away from them. A mark of 1 cm in diameter was stuck on the target, and the archers aimed at this mark each time. This study used one optical camera (Bonita 480 m; Oxford Metrics, Oxford, UK) at 100 Hz to capture the moment of the thrown arrows and record the shoulder joint angle (shoulder abduction and horizontal extension) at that moment (Figure S1). The moment when the arrow was thrown was defined as the moment when the fingers on the string (the drawing side) were straightened. Shooting motion data were captured with an eight-camera system (T20; Oxford Metrics) at 100 Hz. The evaluation was performed using marker sets for modified Plug-In Gait full-body modelling, with the markers attached to the skin with adhesive surgical tape [20]. The data were processed using the Nexus motion analysis system v2.5 (Vicon; Oxford Metrics). The shooting of six arrows was recorded consecutively and the average of the six arrows was taken for analysis.

### 2.5. LT Strength

Participants were provided instructions regarding the test procedure. They lay in the prone position, with the upper extremity diagonally overhead, in line with the fibres of the LT muscle [21]. The participants were then instructed to maintain the arm in the test position while the examiner provided resistance. The handheld dynamometer (MicroFet 2; Hogan Health Industries, West Jordan, UT, USA) force sensor was applied to the distal one-third of the participant’s radial forearm, and a downward force was applied by the examiner until the participant’s maximal muscular effort was overcome. The maximum force on the handheld dynamometer was recorded. Three trials were recorded consecutively on each upper extremity, with a 30 s intertrial rest, and the average for each side was used for analysis.

### 2.6. Shoulder Horizontal Abductor/Adductor Muscle Strength

The shoulder horizontal abductors/adductors muscle strength of the upper limbs was tested with the HUMAC NORM system (CSMi, New York, NY, USA) with the mode of concentric at the 60°/s for horizontal abductor/adductor. All tests were repeated five times, and the maximum strength recorded each test. The participants lay prone and received verbal encouragement during peak torque exertion. Muscle strength was normalised to body weight [22].

### 2.7. Surface Electromyography

Prior to electrode application, the skin was cleaned by being scrubbed with alcohol to reduce skin impedance. Ag/AgCl electrodes (Medi-Trace 200, Covidien/Kendall, USA) with a centre-to-centre distance of 2 cm were placed longitudinally on the muscle belly along the bow and drawing sides upper trapezius (UT), LT, as well as drawing side deltoid middle (DM), deltoid posterior (DP), biceps brachii (BB), and triceps brachii (TB) [8,12]. The reference electrode was placed on the olecranon process. EMG data were amplified (BioNomadix, BIOPAC systems, Goleta, CA, USA) and subjected to analogue/digital (A/D) conversion at 1000 Hz. The data were processed using a Nexus motion analysis system v2.5 (Vicon; Oxford Metrics). We analysed the ratio of LT to UT activity (the UT/LT ratio) as an indicator of elite athletes, which represented a biomechanical factor involving shoulder function [8,12].

EMG activities of all muscles were quantified. EMG data from the six shots were full-wave rectified. The data were then analysed using an integral calculus level of 1 s before the arrows were thrown. Prior to the shots, the maximum voluntary contractions (MVC) of all six muscles were determined. Shot six arrows were recorded consecutively and take the average of the six arrows for analysis.

Manual muscle testing was used to calculate the isolated maximum muscle strength [8,12,23]. Participants performed 3 s maximum voluntary isometric contractions against manual resistance. UT strength was tested in the standing position, and resistance was applied to the scapular elevation. LT strength was tested in the prone position, with the shoulder at 140° flexion and 135° abduction, and resistance was applied to the point between the acromion and scapular spine. DM strength was tested in the standing position with 90° shoulder abduction, and resistance was applied to humeral abduction. DP strength was tested in the prone position with 90° shoulder abduction, and resistance was applied to the horizontal abduction of the humerus. BB strength was tested in the standing position with 0° shoulder flexion and 90° elbow flexion, and resistance was applied to elbow joint flexion. TB strength was tested in the prone position with the shoulder at 0° flexion and 90° abduction and 90° elbow flexion, and resistance was applied to elbow joint flexion. The MVC were performed and measured three times, and the mean values were considered for analysis. After full-wave rectification, EMG amplitudes were normalised to MVC.

### 2.8. Additional Exercise Protocol

Elastic resistance bands can be a viable option to conventional resistance-training equipment during single-joint resistance exercises [24,25]. Elastic band resistance training produces adaptations similar to those with weight resistance training in the early phases of strength training [26]. However, none of the participants had received the LT exercise program. To avoid the possible compensatory effects of using resistance equipment [27], which can reduce the efficiency of LT exercise program, elastic bands (Thera-Band Black, USA) were used for exercise program at the beginning (first 4 weeks), and cable stack machines (Matrix G3-MS50; Johnson Health Tech, Taichung, Taiwan) were used subsequently (Table S2). The LT, latissimus dorsi, rhomboid, middle trapezius, and serratus anterior would be trained in the training program.

In the first 4 weeks, elastic bands were used during the following exercises, which were performed 3 days a week: reverse fly, straight-arm pull-down, reverse straight-arm pull-down, and straight-arm seated row. In addition, the participants performed floor movements, including locust pose and superman pose. Each set included 15 s isometric contractions; each trial had five sets, with a 15 s break between sets.

In weeks 5–12, cable stack machines were used during the following exercises, which were performed 3 days a week: reverse cable fly, straight-arm pull-down, reverse straight-arm pull-down, straight-arm seated row, and Y shape lat pull-down. In addition, alternative isometric contractions in pull-up and pull-down positions were performed for 15 s per set, with five sets per trial and a 15 s rest between sets.

Ten minutes of warm-up and cool-down were performed before and after the exercise program. At least one trained physical coach supervised the motion and physical conditions of the participants during the exercise program.

### 2.9. Statistical Analysis

All data analyses were performed using SPSS for Windows (version 20.0; SPSS, Chicago, IL, USA). All continuous data were presented in terms of mean ± standard deviation. The 2 × 2 (group × time) repeated measures ANOVA was used to analyse intergroup and the between pre-and post-exercise training differences. Use Fisher’s exact test to compare whether there was a difference in the proportion of athletes who have increased or decreased the score of the simulation game between the groups. No abnormal distributions were noticed in pre-tests according to Shapiro–Wilk results for both groups. The relative effect size (ES) for the performance data was calculated using Cohen’s *d*. It is defined as the difference between two means divided by a standard deviation for the data. Additionally, the categorical variables’ ES would use Cohen’s *w.* Intraclass correlation coefficient (ICC), typical error (TE), and percentual coefficient of variation (CV) were calculated to access intertest correlation (pre-and post-exercise training). Significance was set at the level of *p* ≤ 0.05. The power was determined using G*power software version 3.1.9.7. (Heinrich Heine University, Dusseldorf, Germany). The input parameters used for the *F* test were alpha = 0.05 and the primary outcome (simulated game) of effect size, 0.56. The power was calculated as 0.97.

## 3. Results

The 14 athletes (five male and nine female university archers) were divided into exercise and control groups. The mean age of the enrolled athletes was 19 years. No significant intergroup differences were found in demographic characteristics, including age, height, weight, body mass index, and years in archery (*p* > 0.05; Table S3). Furthermore, it was shown that the age, years of training, and score was similar between groups. It means that all athletes’ performance and skill levels were similar [8].

**Figure 1:**
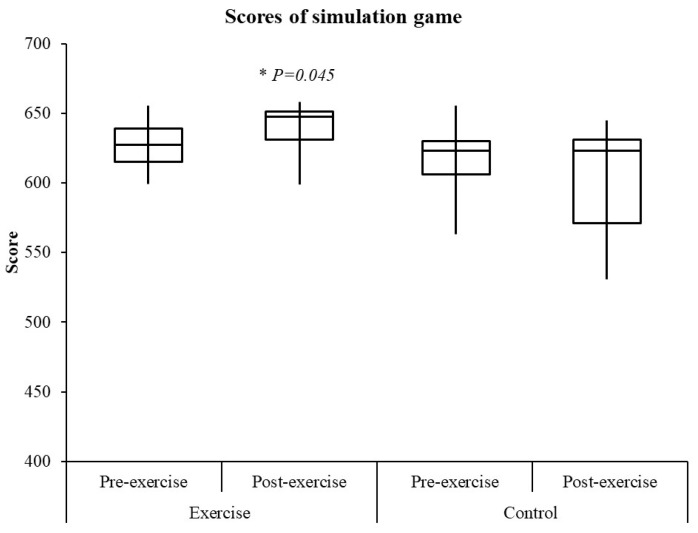
Scores of simulation game. * *p* ≤ 0.05 between pre- and post-exercise.

In the exercise group, the average score of the simulation game increased from 628 ± 19 to 639 ± 20 after the training regimens (*p* = 0.045, Cohen’s *d* = 0.56, ICC = 0.84, CV = 3%, TE = 4; Figure 1). The effect size was 0.56 which belongs to moderate Cohen’s *d* [28,29], the ICC was 0.84 which belongs to good and the CV was 3% [30]. Additionally, a CV of 10% or less was set as the level at which a measure was considered reliable [31]. No such increases were noted in the control group (617± 29 to 601 ± 44, *p* = 0.125, Cohen’s *d* = 0.30). In the final simulation game performance, the score of the exercise group was not significantly higher than that of the control group (*p* = 0.062, Cohen’s *d* = 1.11). However, all athletes in the exercise group have improved their scores in the simulated game after the exercise program. The scores of two athletes in the control group increased. The number of simulated game score increases in the exercise group was significantly higher than that in the control group (*p* = 0.026, Cohen’s *w* = 0.043).

**Figure 2:**
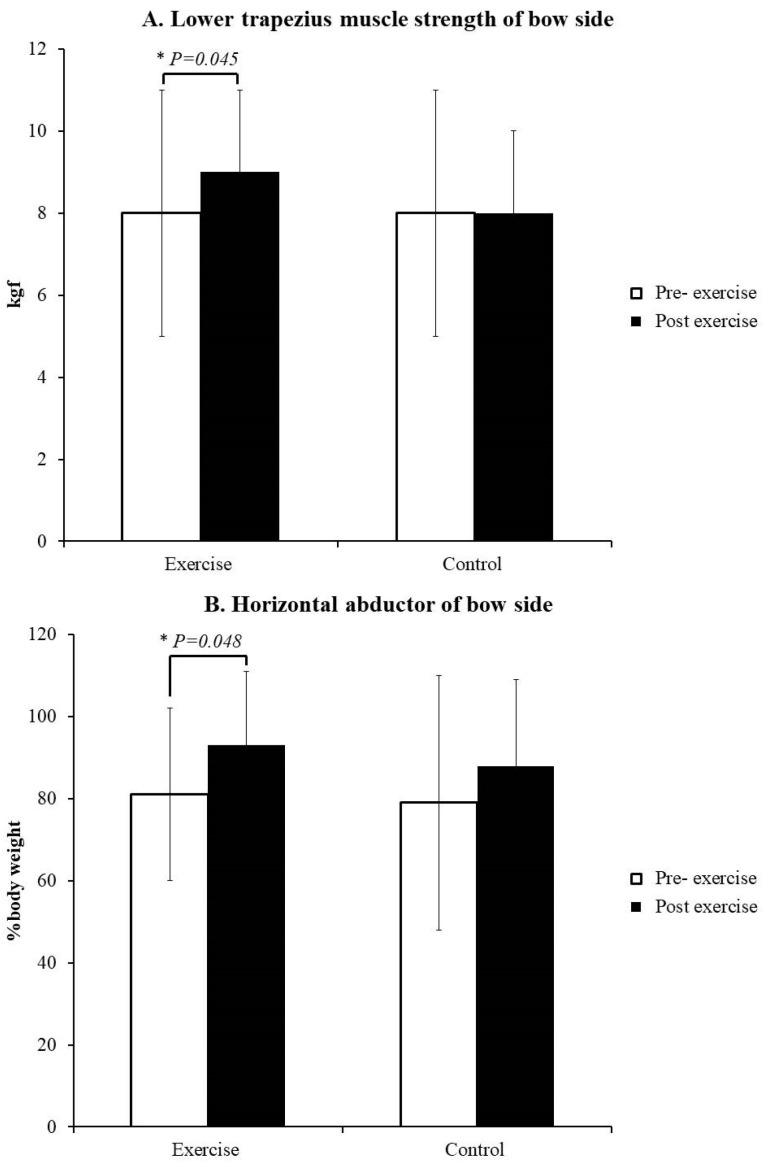
(**A**). Lower trapezius muscle strength of bow side; (**B**). Horizontal abductor of bow side. * *p* ≤ 0.05 between pre- and post-exercise.

**Figure 3:**
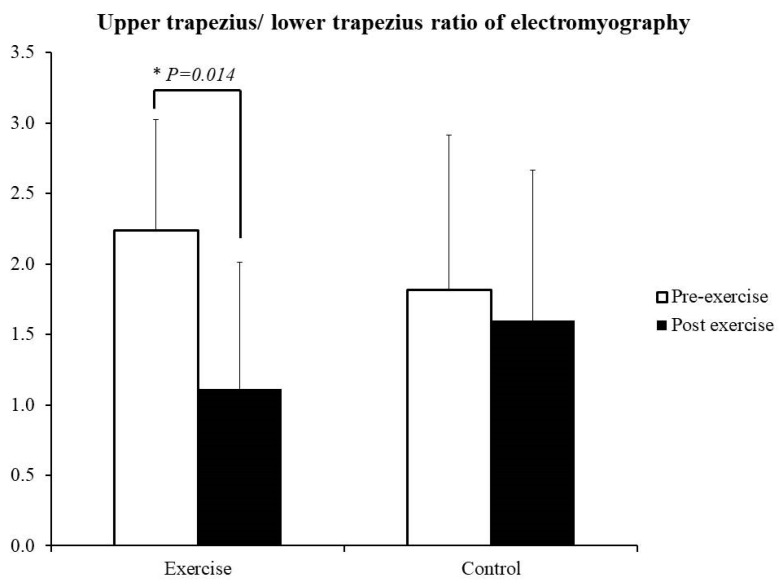
Upper trapezius/lower trapezius ratio as indicated through electromyography. * *p* ≤ 0.05 between pre- and post-exercise.

Regarding muscle strength, LT strength increased from 8 ± 3 to 9 ± 3 kg-force (kgf) after the participants in the exercise group underwent the training regimen (*p* = 0.045, Cohen’s *d* = 0.33, ICC = 0.93, CV = 35%, TE = 2; Figure 2A). Horizontal abductions strength also increased from 81 ± 28 to 93 ± 31 body weight% (*p* = 0.048, Cohen’s *d* = 0.45, ICC = 0.87, CV = 30%, TE = 5; Figure 2B). No such increases were noted in the control group (Table S4). Regarding shoulder kinematics, horizontal extensions and shoulder abduction did not differ between both groups (*p* > 0.05; Table 1). On the drawing side, the activities of UT, BB, and TB decreased after training in the exercise group (*p* ≤ 0.05, Cohen’s *d* ≥ 0.58, ICC ≥ 0.47, CV ≤ 55%, TE ≤ 6; Table 2). No differences in LT were observed. The UT/LT ratio decreased from 2.2 ± 0.8 to 1.1 ± 0.9 after training (*p* = 0.014, Cohen’s *d* = 1.29, ICC = 0.42, CV = 60%, TE = 1; Figure 3). In the control group, only differences in TB were detected on the drawing side.

## 4. Discussion

Notably, the current study revealed that an exercise protocol focusing on LT and other muscles, such as the rhomboid muscles, was associated with an increase in the simulation game score, in conjunction with an increase in LT and horizontal abductor strength. These increases were associated with decreases in the activities of UT, BB, and TB muscles.

Archery requires a high level of stability, which is achieved through the fine coordination of the shoulder girdle muscles. In general, muscle strengthening exercises of the upper extremities usually involve large muscle groups, such as the UT, pectoralis major, anterior deltoid, DP, and latissimus dorsi, to produce gains in strength and hypertrophy. The stabilising muscles, such as LT and serratus anterior, are often overlooked [13]. In addition, hyperactivity and early activation of the UT can lead to excessive scapular elevation when the arm is raised, which impairs performance [32,33,34]. Greater UT activity increases the higher risk of shoulder impingement [12,35,36,37]. Exercises to strengthen the middle trapezius, LT, and serratus anterior muscles can restore muscle balance and improve scapular stability [38,39]. In the present study, we developed a training exercise regimen focusing on the LT-centred muscular that improved the strength of LT and horizontal abductors and, simultaneously, dramatically decreased the muscle activities of UT, BB, and TB in the presence of persistent LT activities. This decrease in muscle activities may enable archers to enhance their stability through being able to meticulous balance themselves.

In the present study, shoulder kinematics did not differ after training. Indeed, scapular protraction and elevation usually result from increased DM and UT activity, along with a reduced activity of the middle trapezius and LT during shoulder abduction [37]. We did not observe decreases in abduction or increases in horizontal extensions. It was possible that the participant’s posture was not modified in this short study period; further instruction to improve their posture should be given. The increased LT strength can aid the fine tuning of the shoulder muscles, such as the deltoid, UT, BB, and TB.

A study advocated focusing on the scapular stabilisers—in particular, the middle trapezius, LT, and serratus anterior—to maintain balance in the scapular force couple [39]. Excessive UT activation with a decreased activation of LT and serratus anterior leads to abnormal scapular movement [32,33,35,36]. The training programme in this study could reduce UT activity during archery and avoid excessive shrug-induced elevation of the shoulder girdle.

The general functions of the trapezius include the following: for the UT, scapular upward rotation and elevation; for the middle trapezius, retraction; and for the LT, upward rotation and depression [40]. In the literature on archery, LT activity is significantly higher among elite players than among pre-elite and beginner players, implying that the LT muscle of the draw arm is actively involved in scapular fixation during shooting [8].

During shoulder abduction, scapular protraction and elevation lead to increased activity of the DM and UT and reduced activity in the middle trapezius and LT [37]. In addition, the inferomedial directed fibres of the LT may also contribute to posterior tilt and the external rotation of the scapula during humeral elevation [41]. However, after training, archery players’ UT activity decreases during archery. This increases the horizontal extension in the aiming stage, whereas shoulder abduction remains unchanged.

In fact, the differences in scores of simulation games could be multifactorial that biomechanics played a partial role. Although the lower trapezius-centred exercise was shown to decrease UT/LT ratio which was favoured biomechanically in the aiming phase, there were still other factors such as mental status/autonomous nervous functions, lower extremities function, and core stability that could contribute to the performance. We further analysed the score results with individual responses, i.e., the number of participants in the exercise and control group had higher scores for post testing, to eliminate the person-related bias. It was found that the number of simulated game score increases in the exercise group was significantly higher than that in the control group. Based on the present study, we suggested that lower trapezius-centred exercise is beneficial biomechanically in the aiming phase. Further interventions such as mindfulness, core muscle training was suggested to further enhance performance.

This study only investigated university archers with small sample size and lack of randomization. In addition, this study uses statistical methods (2 × 2 repeated measures ANOVA) to confirm the baseline data in muscle strength, kinematics, EMG, and game scores were all compared and shown no differences between groups. Additionally, ES, ICC, CV, and TE were used to confirm the reliability of the pre-and post-exercise. In the present study, the additional lower trapezius-centred excise resulted in extra training time that could be partly responsible for the differences between groups. Although this lower trapezius-centred excise was low to medium intensity, further studies in the precise amount of training were suggested. Moreover, the study provided a training model specifically for archers to enhance their performance. Future studies are expected to expand the scope of the investigation and improve the technology involved in archery.

## 5. Conclusions

In conclusion, among archers, the biomechanics of the drawing side affects the performance and thus competition results. The main influencing factor was the strength of the shoulder horizontal abductor at the drawing side. In addition, the designed exercise training regimen can effectively improve competition ranking by increasing shoulder horizontal abductor and LT strength and reducing UT activity.

## Figures and Tables

**Table 1:** Shoulder angle (degree).

			Pre-Exercise	Post-Exercise	ES	ICC	CV(%)	TE
			Mean ± SD	Mean ± SD				
Exercise	Bow side	Shoulder abduction	94 ± 6	91 ± 9	0.39	0.69	8	3
		Horizontal extension	141 ± 20	140 ± 28	0.04	0.91	17	5
	Drawing side	Shoulder abduction	120 ± 4	120 ± 4	0.01	0.76	3	2
		Horizontal extension	147 ± 5	146 ± 5	0.20	0.45	3	2
Control	Bow side	Shoulder abduction	95 ± 5	95 ± 5	0.01	0.61	5	2
		Horizontal extension	130 ± 33	137 ± 21	0.25	0.68	20	5
	Drawing side	Shoulder abduction	123 ± 6	120 ± 4	0.59	0.76	4	2
		Horizontal extension	144 ± 9	143 ± 9	0.11	0.94	6	3

CV: coefficient of variation; ES: effect size; ICC: intraclass correlation coefficient; SD: standard deviation; TE: typical error.

**Table 2:** Before releasing EMG %MVC.

			Pre-Exercise	Post-Exercise	ES	ICC	CV(%)	TE
			Mean ± SD	Mean ± SD				
Exercise	Bow side	Lower trapezius	49 ± 24	42 ± 17	0.33	0.88	44	4
		Upper trapezius	95 ± 35	69 ± 36 *	0.73	0.80	44	6
	Drawing side	Lower trapezius	38 ± 16	40 ± 10	0.15	0.84	33	4
		Upper trapezius	78 ± 22	44 ± 36 *	1.14	0.47	55	6
		Deltoid posterior	85 ± 13	74 ± 15	0.78	0.71	18	4
		Deltoid middle	59 ± 12	59 ± 14	0.01	0.69	21	4
		Triceps brachii	26 ± 10	19 ± 14 *	0.58	0.88	55	4
		Biceps brachii	42 ± 15	29 ± 12 *§	0.96	0.57	41	4
Control	Bow side	Lower trapezius	63 ± 25	52 ± 17	0.51	0.74	37	5
		Upper trapezius	95 ± 17	95 ± 21	0.01	0.48	19	4
	Drawing side	Lower trapezius	54 ± 30	50 ± 24	0.15	0.88	50	5
		Upper trapezius	77 ± 21	65 ± 30	0.46	0.37	36	5
		Deltoid posterior	93 ± 10	88 ± 4	0.66	0.35	8	3
		Deltoid middle	69 ± 16	56 ± 18	0.76	0.32	28	4
		Triceps brachii	34 ± 13	23 ± 12 *	0.88	0.77	46	4
		Biceps brachii	54 ± 32	51 ± 24	0.11	0.97	51	5

CV: coefficient of variation; ES: effect size; ICC: intraclass correlation coefficient; MVC: maximum voluntary contractions; SD: standard deviation; TE: typical error. * *p* ≤ 0.05 between pre- and post-exercise. § *p* ≤ 0.05 between exercise and control group.

## Data Availability

Remaining data are available from the corresponding author upon reasonable request.

## Supplementary Materials

The following are available online at https://www.mdpi.com/2227-9032/10/1/171/s1, Table S1: Routine exercise protocol, Table S2: Additional to routine exercise protocol, Table S3: Participants’ demographic characteristics, Table S4: Muscle strength; Figure S1: Shoulder angle of drawing side.
