# Correction: Liao et al. Twelve-Week Lower Trapezius-Centred Muscular Training Regimen in University Archers. *Healthcare* 2022, *10*, 171

**DOI:** 10.3390/healthcare10020378

**Published:** 2022-02-17

**Authors:** Chien-Nan Liao, Chun-Hao Fan, Wei-Hsiu Hsu, Chia-Fang Chang, Pei-An Yu, Liang-Tseng Kuo, Bo-Ling Lu, Robert Wen-Wei Hsu

**Affiliations:** 1 Department of Athletic Sports, National Chung Cheng University, Chia Yi 621, Taiwan; liao.cody@gmail.com; 2 Sports Medicine Center, Chang Gung Memorial Hospital, Chia Yi 621, Taiwan; fun711009@cgmh.org.tw (C.-H.F.); kojia882@yahoo.com.tw (C.-F.C.); b9002065@cgmh.org.tw (P.-A.Y.); light71829@gmail.com (L.-T.K.); bolin271@yahoo.com.tw (B.-L.L.); wwh@cgmh.org.tw (R.W.-W.H.); 3 Department of Orthopedic Surgery, Chang Gung Memorial Hospital, Chia Yi 621, Taiwan; 4 School of Medicine, Chang Gung University, Tao-Yuan 333, Taiwan

The authors would like to make the following corrections to the published paper [1].

Replacing the Institutional Review Board number 2019800990B0 with 201800990B0.
