# Supplementary material for: Twelve-Week Lower Trapezius-Centred Muscular Training Regimen in University Archers
Source: Healthcare (Basel). 2022 Jan 17;10(1):171. doi: 10.3390/healthcare10010171 (PMC8775688; doi:10.3390/healthcare10010171)
Supplement: Supplementary file 1 [file healthcare-10-00171-s001.zip › Table S1 Routine exercise protocol.pdf]

Table S1. Routine exercise protocol

| Time | Monday | Tuesday | Wednesday | Thursday | Friday | Saturday | Sunday |
|---|---|---|---|---|---|---|---|
| 6:30-8:00 | Addition to routine exercise protocol for exercise group | | Addition to routine exercise protocol for exercise group | | Addition to routine exercise protocol for exercise group | Rest | Rest |
| 8:30-11:30 | 1. Short-range archery × 150 sticks<br>2. 70 m × 108 sticks | 1. Simulation game × 2 (individual race) | 1. Simulation game (individual race) | 1. Short-range archery × 150 sticks<br>2. 70 m × 144 sticks | Self-adjustment | Self-adjustment | Rest |
| 13:30- End of training | 1. Short-range archery × 150 sticks<br>2. 70 m × 144 sticks<br>3. Jogging for 2 km | 1. Short-range archery × 150 sticks<br>2. Resistance training<br>3. Jogging for 2 km | 1. Simulation game (team competition) | 1. Short-range archery × 150 sticks<br>2. Simulation game (individual race)<br>3. Jogging for 2 km | Self-adjustment | Rest | Rest |

Self-adjust: Training conducted according to the participant's individual needs (e.g., archery at 70 m, resistance training, and jogging).

Resistance training:

1. Two-handed dumbbell curl, two-handed dumbbell shoulder press, upright pull dumbbell, dumbbell one-arm rowing, barbell press, barbell squat. All dumbbell exercises: 15 reps, 3 sets.
2. Push-ups × 25, bend abdomen × 25, unilateral foot lift × 25, burpee × 25, bear crawl × 10 m.
3. Maintain the bowstring for 20 s × 6.
