# Supplementary material for: Twelve-Week Lower Trapezius-Centred Muscular Training Regimen in University Archers
Source: Healthcare (Basel). 2022 Jan 17;10(1):171. doi: 10.3390/healthcare10010171 (PMC8775688; doi:10.3390/healthcare10010171)
Supplement: Supplementary file 1 [file healthcare-10-00171-s001.zip › Table S2 Additional to routine exercise protocol.pdf]

Table S2. Additional to routine exercise protocol

Period	Name	Frequency	Intensity	Time	Type
1-4 weeks	Reverse fly	3 days a week	Elastic bands (Thera-Band Black)	15 s per set, 5 sets per trial, with a 15-s rest between sets.	Isometric contractions
	Straight-arm pull-down		Elastic bands (Thera-Band Black)		
	Reverse straight-arm pull-down		Elastic bands (Thera-Band Black)		
	Straight-arm seated row		Elastic bands (Thera-Band Black)		
	Locust pose		Weight bearing		
	Floor superman pose		Weight bearing		
5–12 weeks	Reverse cable fly	3 days a week	60% 1 RM	15 repetitions per set, 5 sets per trial, with a 15-s rest between sets.	Resistance training
	Straight-arm pull-down		60% 1 RM		
	Reverse straight-arm pull-down		60% 1 RM		
	Straight-arm seated row		60% 1 RM		
	Straight-arm seated row and Y shape lat pull-down		60% 1 RM		
	Pull-ups		Weight bearing	15 s per set, 5 sets per trial, with a 15-s rest between sets.	Isometric contractions
	Pull-down		Weight bearing		
