# Supplementary material for: Twelve-Week Lower Trapezius-Centred Muscular Training Regimen in University Archers
Source: Healthcare (Basel). 2022 Jan 17;10(1):171. doi: 10.3390/healthcare10010171 (PMC8775688; doi:10.3390/healthcare10010171)
Supplement: Supplementary file 1 [file healthcare-10-00171-s001.zip › Table S3 Participantsí» demographic characteristics.pdf]

Table S3. Participants' demographic characteristics

	Exercise group (M = 3; F = 4)		Control group (M = 2; F = 5)	
	Mean	± SD	Mean	± SD
Age (year)	19	± 1	19	± 1
Height (cm)	169	± 9	165	± 6
Weight (kg)	73	± 13	67	± 13
BMI	25	± 3	24	± 5
Years of training (year)	8	± 1	6	± 1

M: Male; F: Female; BMI: body mass index；SD: standard deviation
