# Supplementary material for: Twelve-Week Lower Trapezius-Centred Muscular Training Regimen in University Archers
Source: Healthcare (Basel). 2022 Jan 17;10(1):171. doi: 10.3390/healthcare10010171 (PMC8775688; doi:10.3390/healthcare10010171)
Supplement: Supplementary file 1 [file healthcare-10-00171-s001.zip › Table S4 Muscle strength.pdf]

Table S4. Muscle strength

			Pre-exercise		Post-exercise		ES	ICC	CV(%)	TE
			Mean	± SD	Mean	± SD				
Exercise	Bow side	Lower trapezius (kgf)	8	± 3	9	± 3 *	0.33	0.93	35	2
		Horizontal abductor (BW%)	81	± 21	93	± 31 *	0.45	0.87	30	5
		Horizontal adductor (BW%)	81	± 28	83	± 30	0.07	0.99	34	5
	Drawing side	Lower trapezius (kgf)	9	± 3	9	± 3	0.01	0.91	33	2
		Horizontal abductor (BW%)	86	± 19	93	± 26	0.31	0.93	25	5
		Horizontal adductor (BW%)	79	± 30	83	± 36	0.12	0.97	39	6
Control	Bow side	Lower trapezius (kgf)	8	± 2	8	± 2	0.01	0.97	23	1
		Horizontal abductor (BW%)	79	± 18	88	± 21	0.46	0.87	23	4
		Horizontal adductor (BW%)	81	± 18	77	± 22	0.20	0.92	25	4
	Drawing side	Lower trapezius (kgf)	8	± 2	7	± 2	0.50	0.87	27	1
		Horizontal abductor (BW%)	88	± 13	88	± 17	0.01	0.92	17	4
		Horizontal adductor (BW%)	85	± 15	81	± 24	0.20	0.83	23	4

BW: body weight; CV: coefficient of variation; ES: effect size; ICC: intraclass correlation coefficient; Kgf: kilogram-force; SD: standard deviation; TE: typical error

* $P \leq 0.05$ between pre- and post-exercise
