# Supplementary figures and images for: Twelve-Week Lower Trapezius-Centred Muscular Training Regimen in University Archers
Source: Healthcare (Basel). 2022 Jan 17;10(1):171. doi: 10.3390/healthcare10010171 (PMC8775688; doi:10.3390/healthcare10010171)

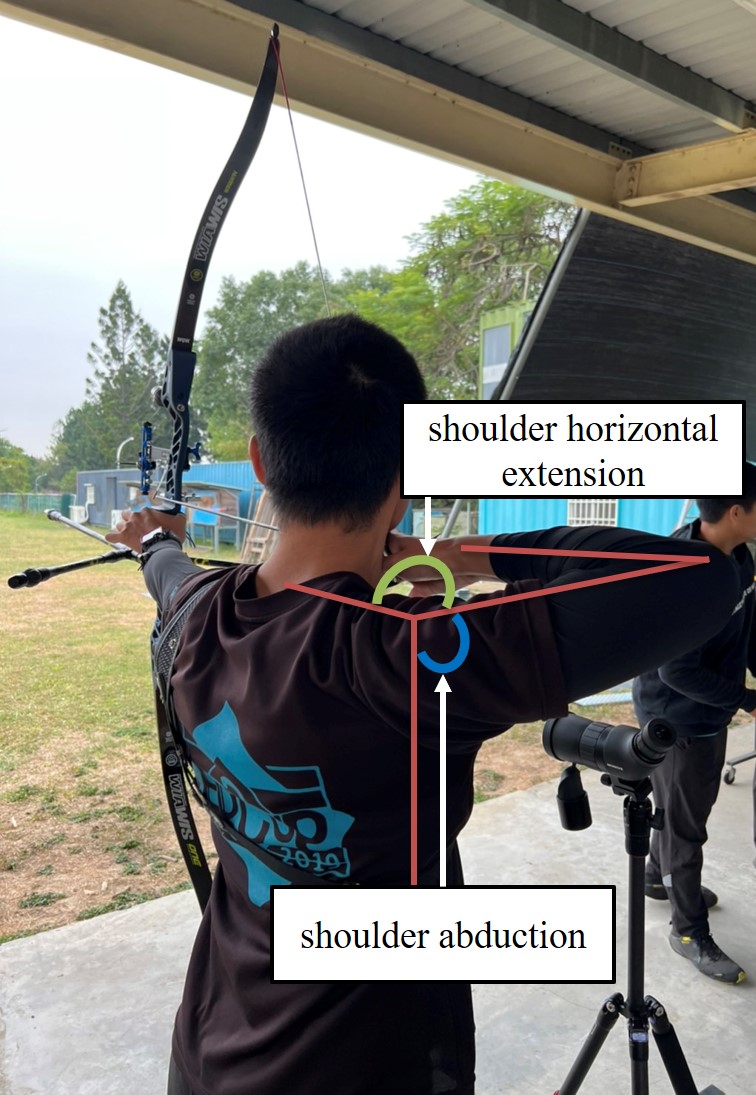

Supplement: Supplementary file 1 [file healthcare-10-00171-s001.zip › Figure S1.jpg]
